# Devices for *In situ* Development of Non-disturbed Oral Biofilm. A Systematic Review

**DOI:** 10.3389/fmicb.2016.01055

**Published:** 2016-07-19

**Authors:** Isabel Prada-López, Víctor Quintas, Carlos Vilaboa, David Suárez-Quintanilla, Inmaculada Tomás

**Affiliations:** ^1^Oral Sciences Research Group, Special Needs Unit, School of Medicine and Dentistry, Universidade de Santiago de CompostelaLa Coruña, Spain; ^2^Dental Prosthesis Laboratory, School of Medicine and Dentistry, Universidade de Santiago de CompostelaLa Coruña, Spain

**Keywords:** biofilm, dental plaque, device design, *in situ*, splints

## Abstract

**Objective:** The aim of this review was to assess the types of devices used for *in situ* development of oral biofilm analyzed microbiologically.

**Materials and Methods:** A systematic search of the literature was conducted to identify all *in situ* studies of oral biofilm which used an oral device; the Ovid MEDLINE and EMBASE databases complemented with manual search were used. Specific devices used to microbiologically analyze oral biofilm in adults were included. After reading of the selected full texts, devices were identified and classified according to the oral cavity zone and manufacturing material. The “ideal” characteristics were analyzed in every group.

**Results:** The search provided 787 abstracts, of which 111 papers were included. The devices used in these studies were classified as palatal, lingual or buccal. The last group was sub-classified in six groups based on the material of the device. Considering the analyzed characteristics, the thermoplastic devices and the Intraoral Device of Overlaid Disk-holding Splints (IDODS) presented more advantages than limitations.

**Conclusions:** Buccal devices were the most commonly used for the study of *in situ* biofilm. The majority of buccal devices seemed to slightly affect the volunteer's comfort, the IDODS being the closest to the “ideal” model.

**Clinical Relevance:** New devices for *in situ* oral biofilm microbiological studies should take into account the possible effect of their design on the volunteer's comfort and biofilm formation.

## Introduction

The oral cavity contains hard as well as soft tissue surfaces, all of which are potentially available as susceptible areas for the development of oral biofilms (Newman and Wilson, 1999). A specialized model of oral biofilm is dental plaque, which has been defined as a community of microorganisms found on the tooth surface as a biofilm, embedded in a matrix of polymers of salivary and bacterial origin (Marsh, 1999).

Biofilms are important, because some resident species contribute to the maintenance of oral health and other species have the potential to cause local or systemic disease (Newman and Wilson, 1999). In fact, they are involved in the appearance or evolution of most oral conditions, such as caries and decalcifications, periodontal diseases or halitosis (Bowden and Li, 1997). In addition, they have direct impact on the regeneration and tissue healing after oral manipulation (Biofilm Club and Gilbert, 2007). Accordingly, a better knowledge of the oral biofilm characteristics results in the development of better strategies which are more effective in oral disease management (Arweiler et al., 2004). Apart from this, a study of the oral biofilm in individuals with systemic disease could help with the establishment of cause-effect relationships between dental plaque and specific systemic pathologies of possible oral origin (Li et al., 2000).

The creation of *in vitro* biofilm models has contributed to significant advances in the study of oral diseases (Jefferson and Cerca, 2006; Kolenbrander et al., 2006; McBain, 2009; Nobbs et al., 2009; Palmer, 2010). However, their known limitations have caused the scientific community to recognize that the *in vitro* models might not generate a biofilm comparable to those found *in situ* (Wecke et al., 2000; Auschill et al., 2004, 2005; Watson et al., 2005; Al-Ahmad et al., 2007). For this reason, *in vitro* results must be interpreted cautiously (Auschill et al., 2004; Al-Ahmad et al., 2007; Hannig and Hannig, 2009). This affirmation establishes the need to develop models of biofilm *in situ* which could be analyzed *ex vivo* without distortion (Costerton et al., 1999; Palmer et al., 2001; Auschill et al., 2004; Hannig and Hannig, 2009).

In several *in situ* biofilm studies, the sample was recollected from the tooth surface for analysis with paper points (Charles et al., 2000), cotton rolls (Rosin et al., 2002) or scalers (Pan et al., 2000; Daneshmand et al., 2002; Konig et al., 2002; Fine et al., 2005; Loivukene et al., 2005; Arweiler et al., 2006; van der Mei et al., 2006; Al-Ahmad et al., 2010b). These procedures potentially disturb the delicate three-dimensional relationship between cells, the extracellular matrix and the substrate (Wecke et al., 2000; Wood et al., 2000; Dige et al., 2009a). This relation directly influences the biofilm behavior (Wood et al., 2000), which implies that a “non-disturbing” methodology must be applied in the study of any antimicrobial agent (Wood et al., 2000; Beyth et al., 2010). A “non-disturbing” methodology means that the biofilm is not altered during its formation, recollection, processing or analysis. In the literature, numerous artificial substrates were used instead of the natural surface of the teeth in order not to disturb the dental plaque at any stage of analysis; the resultant biofilm is known as Plaque-Like Biofilm (PL-Biofilm) (García-Caballero et al., 2013; Tomás et al., 2013; Quintas et al., 2015b).

The literature defines several specific devices which were designed to form a PL-Biofilm *in situ*. In history, the first papers involving the use of devices for the formation of PL-Biofilm studied the decay, analyzing the demineralization effect (Ahrens, 1976; Koulourides et al., 1976; Ostrom and Koulourides, 1976; Minah and Chu, 1984). Some of these studies used the volunteer's own prosthesis (Koulourides et al., 1976; Minah and Chu, 1984) or their orthodontic appliances (Ostrom and Koulourides, 1976; Jongsma et al., 2015) as artificial substrates for evaluating the oral biofilm activity. On the contrary, Ahrens (1976) designed a specific device in order to study this phenomenon. Later, in 1987, Nyvad et al. (Nyvad and Fejerskov, 1987a,b; Nyvad and Kilian, 1987) analyzed the characteristics of the *in situ* biofilm, formed on the device designed by Ahrens. Since then, the development of these devices has not stopped, but the format has changed from a bulky and poor esthetic to a discreet and comfortable one. One of the last devices to be designed was the “Intraoral Device of Overlaid Disk-holding Splints” (IDODS) (García-Caballero et al., 2013; Tomás et al., 2013; Quintas et al., 2015b), which uses a thermoplastic material with the intention of interfering as little as possible with the normal life of the volunteers. This evolutionary process left many different devices without any standardization or control. A deep analysis of the characteristics of each type of apparatus could help investigators in the field to choose one or another, depending on the aim of their study. In addition, the presentation of the data as advantages and disadvantages of each device could encourage the scientific community in the development of new devices, eventually reaching the “ideal” model. For these reasons, a systematic review of the quality and functionality of the different devices is proposed. The aim of this review was to assess, in adult population, the types of devices used for *in situ* development of oral biofilm analyzed microbiologically.

## Materials and methods

A systematic review protocol was made in the planning stages according to the PRISMA checklist and approved by all authors. This review is reported according the PRISMA statement (Liberati et al., 2009). The PRISMA Checklist is attached as Table S1 in Supplementary Material.

### Focused question

This was the Patient/Population Intervention Comparison Outcome (PICO) question: In adult population, what are the advantages and disadvantages of the different types of devices that have been used for the growing of *in situ* oral biofilm?

The components of the PICO were:

Population: all adult volunteers (over 18 years old) wearing intraoral devices.
Intervention: type of device used for the growing of *in situ* oral biofilm.
Comparisons: between different types of devices.
Outcomes: advantages and disadvantages of each of the different devices.

### Eligibility criteria

All types of *in situ* studies on oral biofilm using a specific device (excluding prosthesis or orthodontic appliances) for its growth were considered eligible. The objective was to evaluate studies which took into account the characteristics of a non-disturbed biofilm of more than 4 h of maturation, analyzing its microbiological aspects (such as viability, thickness, structure or bacterial composition). Because of this, any studies which used devices to analyze specific actions of the biofilm on the tooth, such as demineralizations or decay, were excluded. Additionally, those which measured only biochemical aspects (fluoride concentration, pH, etc.) in the oral biofilm were also excluded. The search was limited to humans and *in vivo* or *in situ* studies. No language restrictions were included.

### Information sources and search strategy

The literature search for relevant articles was conducted using the electronic database OVID MEDLINE and OVID EMBASE, the date of the last update was 16th of June 2015. The search strategy included the following search words:

- MeSH terms in all trees/subheadings: “dental plaque,” “biofilms,” and “splints.”
- Keywords for dental plaque and biofilm: “dental plaque,” “dental deposit^*^,” “biofilm^*^,” “biofouling,” and “oral ADJ bacteria.”
- Keywords for splints: “appliance^*^,” “stent^*^,” “splint^*^,” “ferule^*^,” “device^*^,” “apparatus,” “mechanism^*^,” and “gadget^*^.”

The same search strategy was used in the OVID EMBASE database, adapting the MeSH terms. Manual search was done by the reviewers after checking the reference lists of the relevant studies.

### Study selection, data collection process, and data items

Study selection was conducted independently by two reviewers (IP-L and VQ) in the following stages: (1) initial screening of potentially suitable titles and abstracts meeting the inclusion criteria and (2) screening of the full texts identified as possibly relevant in the initial screen. The assistance of translators was sought for studies that were not in English.

Data were extracted using predefined data extraction forms including type of device, localization, substrate, number of participants, biofilm age, microbiological technique to analyze the biofilm, volunteer experience and removal and retention of the substrate.

Disagreements between reviewers were solved through discussion and consensus Kappa index at the first stage was 0.93 and 0.90 at the second stage. A difficulty in the systematic review was the poor description of a device or the absence of correlation between the description and the photographs presented. When doubts appeared, author contact was required. In studies where author contact was not successful and it was not possible to achieve an agreement in the type of device used, the decision was the exclusion.

### Summary measures and synthesis results

After reading the full text, descriptive summary analyses were reported, following systematic review guidelines (Mulrow et al., 1997). Because of the nature of this review a meta-analysis was not performed. The selected papers were classified in regard to the type of device used. The different apparatus found were divided into three categories, according to their design: palatal devices, lingual devices and buccal devices. This classification responds to the zone in which the device was placed within the oral cavity. In buccal devices, a second subdivision was made according to their material. As a result, six buccal device groups were obtained: Acrylic Device (AcD), Leeds *in situ* Device (LiD), Acrylic and Metal Device (AcMD), Metal Device (MD), Thermoplastic Device (TPD) and Intraoral Device of Disk-holding Splints (IDODS). All of the papers which used a device made completely from acrylic were classified in the “AcD group.” Papers which used a device bonded directly to the tooth formed the “LiD group”; those which used a device made of metal and acrylic were classified into the “AcMD group”; when a device made completely of metal was used, they were grouped as “MD group.” Studies including a device made of one thermoplastic sheet were classified in the “TPD group” and, finally, those devices made of two sheets of thermoplastic material formed the “IDODS group.”

Fourteen important qualities and characteristics of the ideal device were standardized by the authors for the analysis of each apparatus. The first eight questions were focused on the technical characteristics, the next three questions investigated the influence of the device on the volunteer's comfort and the final three questions were about manufacturing, placement and economic cost (Table 1).

**Table 1:** **The fourteen characteristics of the ideal “biofilm ***in situ*** device” classified in three main dimensions: technical, volunteer's comfort and economic**.

**TECHNICAL DIMENSION**	
1	Teeth pre-treatment is not necessary
2	Specific teeth are not necessary
3	No accidental unsticking
4	Allows eating
5	Easy withdrawal by the volunteer
6	Easy withdrawal of the sample
7	No contact with cheek /tongue
8	Allows salivary flow through the splint
**VOLUNTEER'S COMFORT DIMENSION**	
9	Allows good oral hygiene
10	Good aesthetic
11	Little bulky
**ECONOMIC DIMENSION**	
12	Adaptable on the 1st appointment
13	Easy placement at 1st time
14	Inexpensive material

## Results

### Study selection

**Figure 1:**
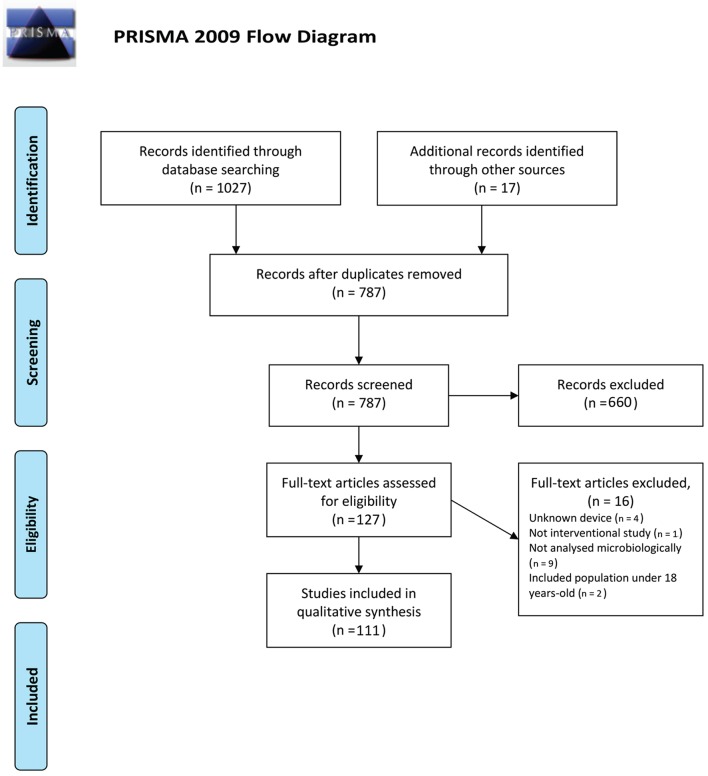
**PRISMA 2009 Flow Diagram**.

The degree of agreement between reviewers was more than 97% at the first and second stages. After the initial search and removing duplications, 787 papers were found. When the titles and abstracts were read, the reviewers selected 127 papers. Following text screening, 16 papers were excluded due to the use of the patient's prosthesis or orthodontic devices, lack of information about the device or other factors, such as not analyzing the biofilm microbiologically or including population of less than 18 years of age. Finally, 111 papers were selected for assessment of the full text (Figure 1, PRISMA flow Diagram).

All of these were written in English, with the exception of one which was in Chinese.

### Study characteristics

All characteristics of the selected studies are in the Data Sheet 1 in Supplementary Material. Following, there is a description of the most noticeable characteristics.

#### Types and number of substrates

The most commonly used substrates for the development of *in situ* oral biofilm have been the human (Creanor et al., 1986; Strassler et al., 1986; Nyvad and Fejerskov, 1987a,b; Nyvad and Kilian, 1987; Jenkins et al., 1988; Nyvad and Fejerskov, 1989; Macpherson et al., 1990, 1991; Robinson et al., 1997; Wood et al., 1999, 2000, 2002; Arai et al., 2000; Cury et al., 2001; Shore et al., 2001; Palmer et al., 2003; Kato et al., 2004; Pecharki et al., 2005; Ribeiro et al., 2005; Diaz et al., 2006; Korytnicki et al., 2006; Robinson et al., 2006; Arthur et al., 2007; Chalmers et al., 2007; Paes Leme et al., 2008; Gameiro et al., 2009; de Mazer Papa et al., 2010; von Ohle et al., 2010; Brambilla et al., 2012; Cochrane et al., 2012; Kato et al., 2012; Teixeira et al., 2012; Pierro et al., 2013) and bovine enamels (Strassler et al., 1986; Hannig, 1997, 1999a,b; Giertsen et al., 2000; Auschill et al., 2001; Jentsch et al., 2002; Tenuta et al., 2003; Paes Leme et al., 2004; Al-Ahmad et al., 2007, 2009, 2010a,c, 2013; Hannig et al., 2007b, 2013b,c; Arweiler et al., 2008; Jung et al., 2010; Brighenti et al., 2012; Kensche et al., 2013; Melo et al., 2013; Arweiler et al., 2014; Bittar et al., 2014; Grychtol et al., 2014) (in this order). Moreover, the number of substrates used in the different devices varied between one (Auschill et al., 2001) and 16 (Hannig, 1997, 1999a,b).

#### Biofilm age

This went from 4 h (Nyvad and Fejerskov, 1987a,b; Nyvad and Kilian, 1987; Zucchelli et al., 1998; Fine et al., 2000; Zucchelli et al., 2000; Diaz et al., 2006; Palmer et al., 2003; Beyth et al., 2010; Claro-Pereira et al., 2011; Kensche et al., 2013; Sreenivasan et al., 2004) to 8 weeks (Benelli et al., 1993; He et al., 2013).

#### Microbiological techniques used for *in situ* PL-biofilm analysis

The most commonly used techniques in these studies are those based on visualizing the oral biofilm with fluorescence microscopes [both epifluorescence and Confocal Laser Scanning Microscope (CLSM)] (Benelli et al., 1993; Netuschil et al., 1998; Wood et al., 1999, 2000, 2002; Giertsen et al., 2000; Auschill et al., 2001, 2002, 2004, 2005; Palmer et al., 2003; Arweiler et al., 2004; Al-Ahmad et al., 2007, 2009, 2010a,c, 2013; Chalmers et al., 2007; Dige et al., 2007, 2009a,b; Arweiler et al., 2008; Hannig et al., 2013a,b,c; Beyth et al., 2010; Burgers et al., 2010; Dong et al., 2010; Gosau et al., 2010; Jung et al., 2010; von Ohle et al., 2010; Bremer et al., 2011; Gu et al., 2012; Rupf et al., 2012; García-Caballero et al., 2013; He et al., 2013; Kensche et al., 2013; Tomás et al., 2013; Arweiler et al., 2014; Grychtol et al., 2014; Padovani et al., 2015; Prada-López et al., 2015a,b; Quintas et al., 2015a,b) (45 studies), mainly combined with fluorescence *in situ* hybridization (FISH) and 4′,6-diamidino-2-phenylindole (DAPI) for bacterial identification. In the case of studies aiming for the analysis of bacterial viability, fluorescence microscopes have usually been combined with staining dyes for live/dead bacterial identification such as SYTO 9/Propidium Iodide and Fluorescein Diacetate/Etidium Bromide.

Another common technique has been the Colony Forming Units (CFU) counting (Creanor et al., 1986; Strassler et al., 1986; Nyvad and Kilian, 1987; Jenkins et al., 1988; Macpherson et al., 1990, 1991; Benelli et al., 1993; Leonhardt et al., 1995; Robinson et al., 1997; Fine et al., 2000; Giertsen et al., 2000; Cury et al., 2001; Shore et al., 2001; Hara et al., 2003; Tenuta et al., 2003; Paes Leme et al., 2004; Sreenivasan et al., 2004; Pecharki et al., 2005; Ribeiro et al., 2005; Korytnicki et al., 2006; Schwarz et al., 2006, 2009; Arthur et al., 2007; Sennhenn-Kirchner et al., 2007; Gameiro et al., 2009; Lima et al., 2009; Sennhenn-Kirchner et al., 2009; Sousa et al., 2009; Sreenivasan et al., 2009; Al-Ahmad et al., 2010a; de Mazer Papa et al., 2010; Jung et al., 2010; von Ohle et al., 2010; Claro-Pereira et al., 2011; Brighenti et al., 2012; Teixeira et al., 2012; Melo et al., 2013; Pierro et al., 2013; Grychtol et al., 2014) (40 studies).

#### Classification of the papers according to the devices

**Figure 2:**
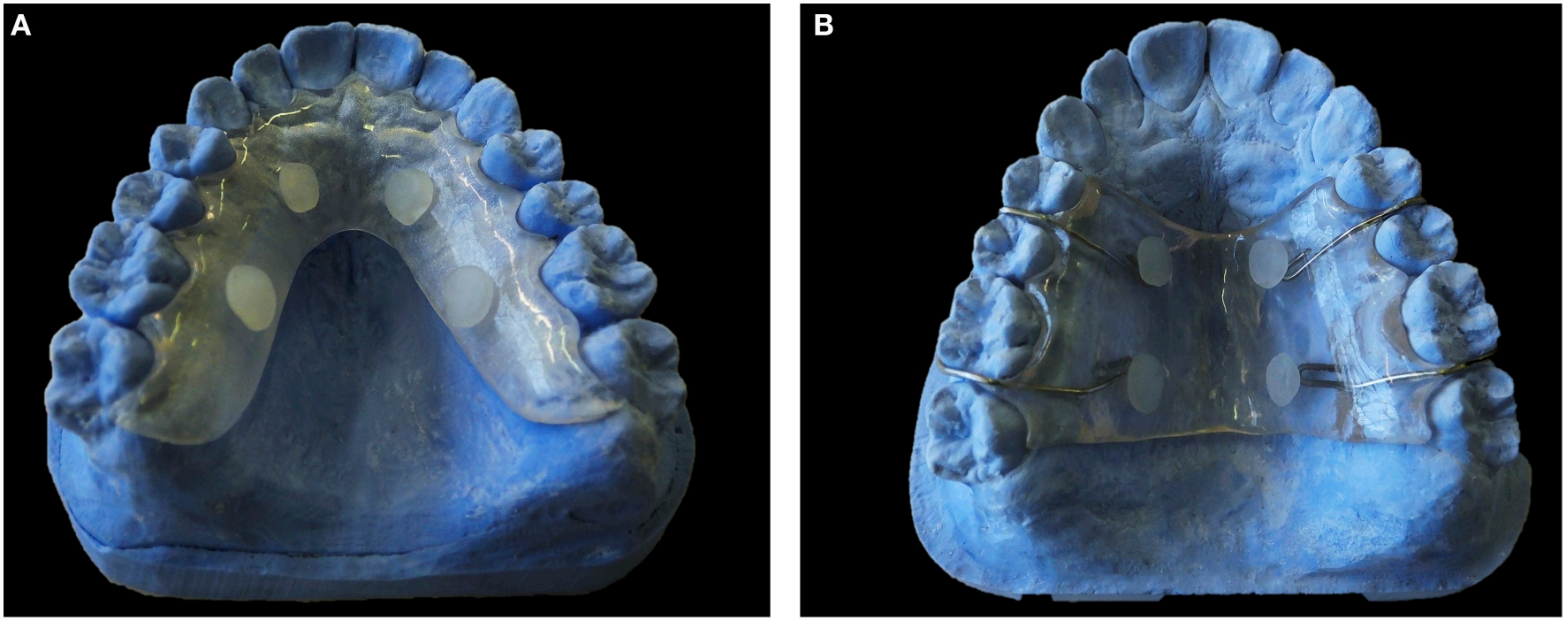
**Palatal Devices. (A)** Model of acrylic palatal device. **(B)** Model of acrylic and metal palatal device.

**Figure 3:**
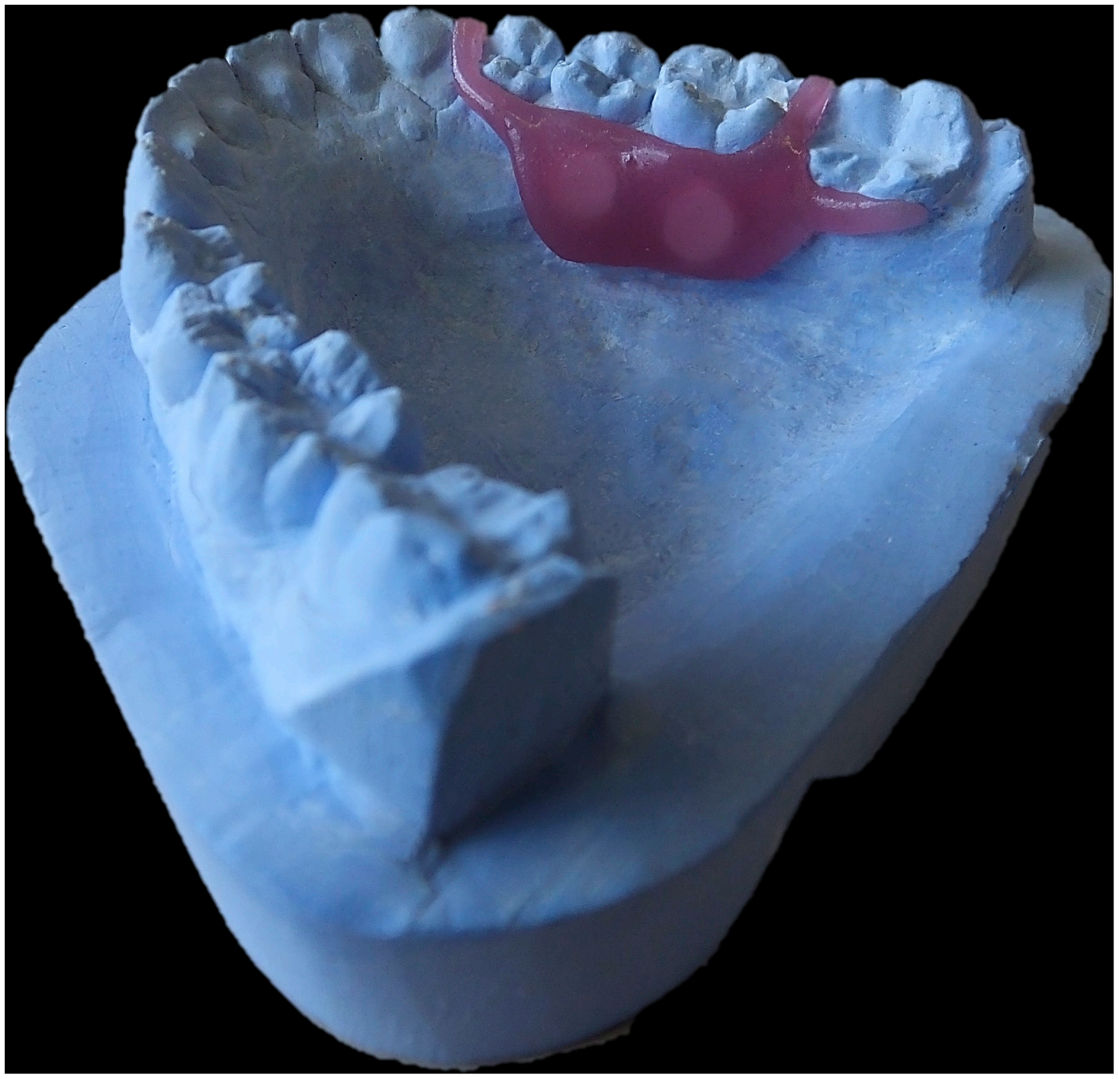
**Lingual Device**. Model of an acrylic lingual device.

Palatal devices (Figures 2A,B; Benelli et al., 1993; Cury et al., 2001; Hara et al., 2003; Tenuta et al., 2003; Paes Leme et al., 2004; Pecharki et al., 2005; Ribeiro et al., 2005; Korytnicki et al., 2006; Schwarz et al., 2006, 2007, 2009; Arthur et al., 2007; Paes Leme et al., 2008; Gameiro et al., 2009; Lima et al., 2009; Sousa et al., 2009; Beyth et al., 2010; de Mazer Papa et al., 2010; Brighenti et al., 2012; Cochrane et al., 2012; Teixeira et al., 2012; Melo et al., 2013; Pierro et al., 2013; Bittar et al., 2014; Padovani et al., 2015) were always exposed to contact with the tongue. To avoid this situation some authors included a plastic mesh (Cury et al., 2001). In lingual devices (Figure 3) (Creanor et al., 1986; Jenkins et al., 1988; Macpherson et al., 1990, 1991; Rasperini et al., 1998; Sreenivasan et al., 2004; Re et al., 2011), the biofilm grew between the device and the lingual gingiva. This protection against tongue contact produced a biofilm that grew in a different environment from the normal lingual biofilm attached to the surface of the teeth. The use of these devices has not been very extensive (only seven studies used them).

**Figure 4:**
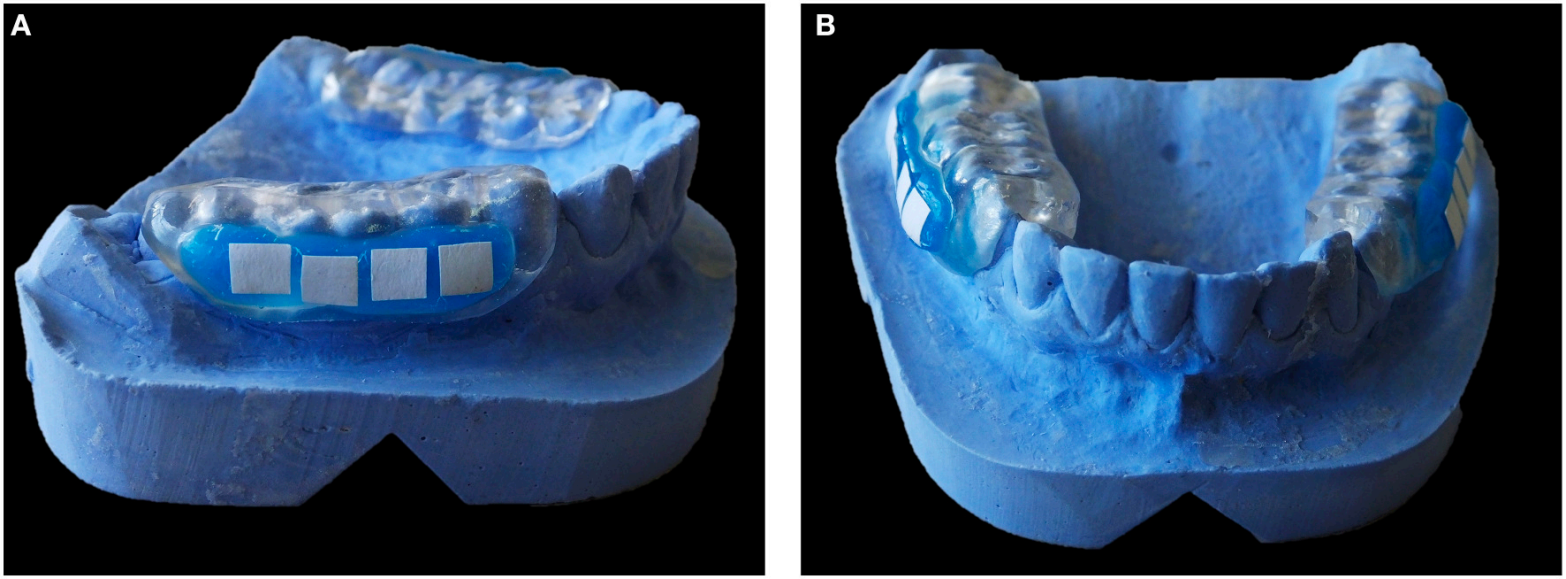
**Acrylic Device (AcD). (A)** Lateral view. **(B)** Frontal view.

**Figure 5:**
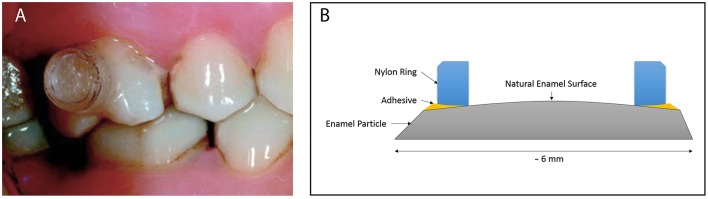
**Leeds ***in situ*** Device (LiD). (A)** Intraoral image by Pessan et al. (2008) in Journal Appliance Oral Science. **(B)** Own design scheme.

**Figure 6:**
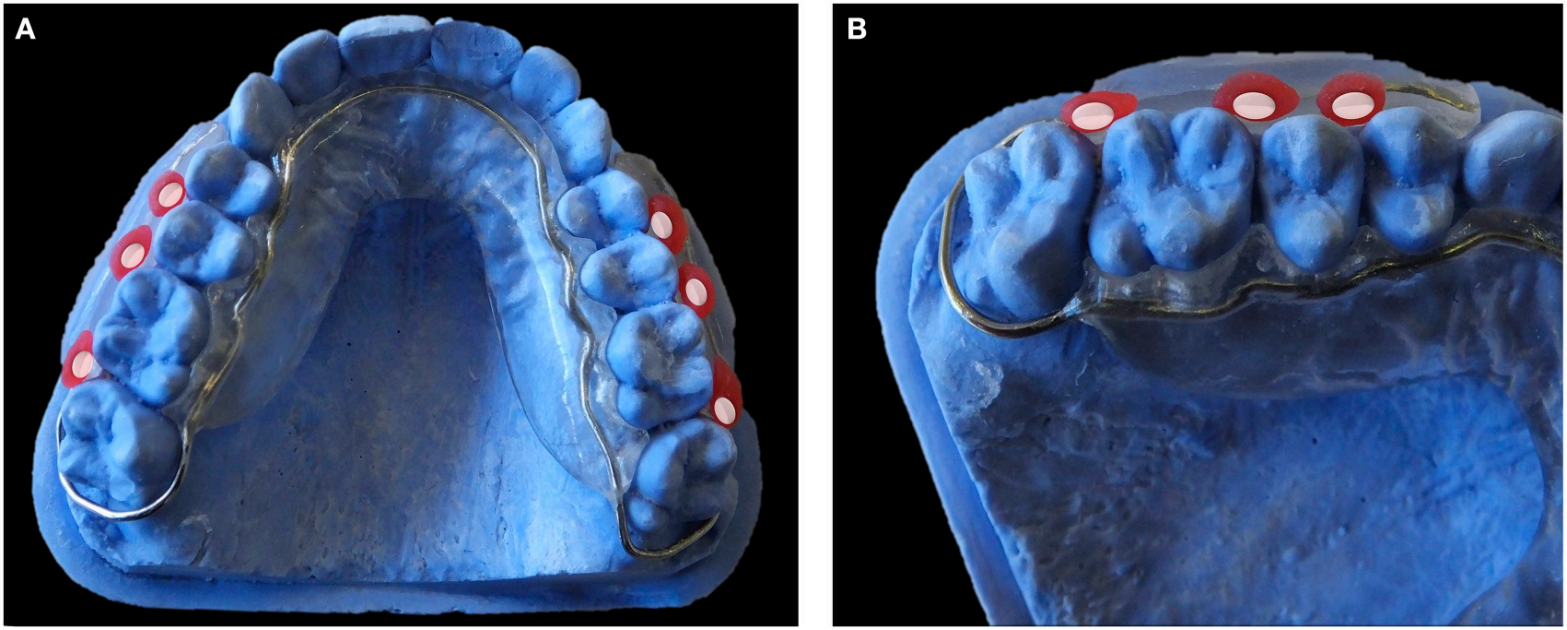
**Acrylic and metal device (AcMD). (A)** Occlusal view. **(B)** Detail of the disk zone.

**Figure 7:**
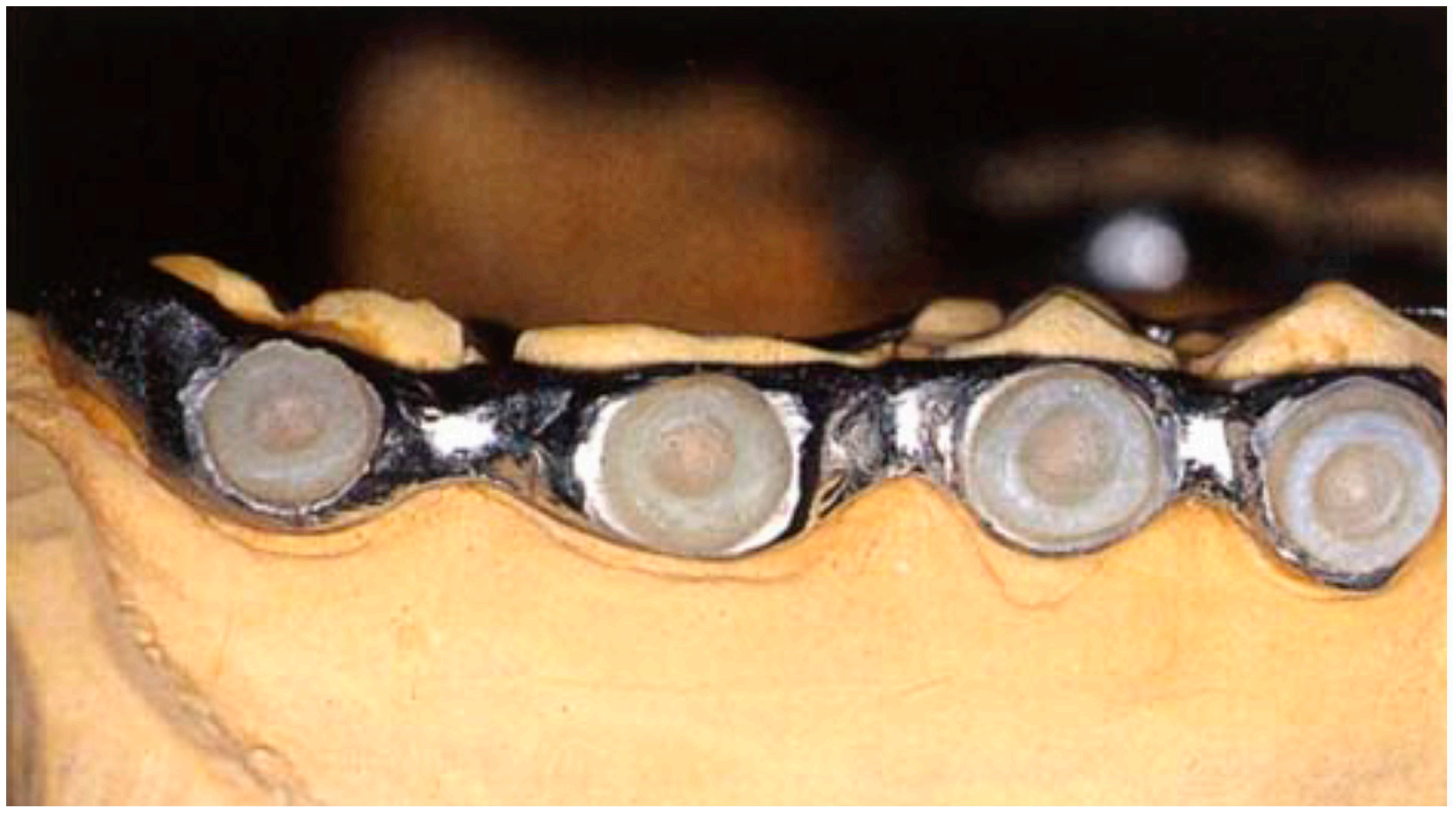
**Metal Device (MD)**. Image by Simion et al. (1997) in Clinical Oral Implants Research.

**Figure 8:**
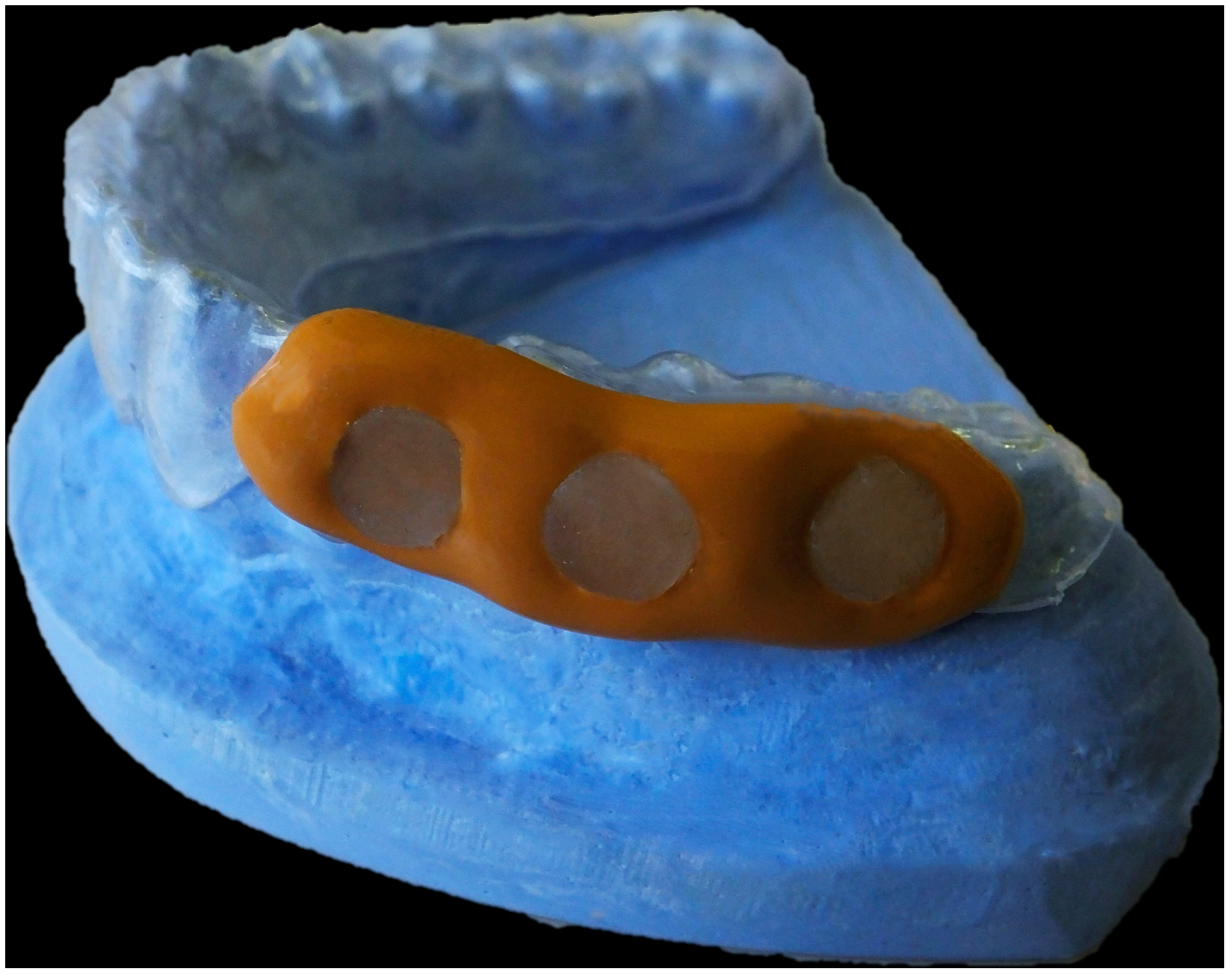
**Thermoplastic Device (TPD)**. Model of a thermoplastic and polysiloxane splint.

**Figure 9:**
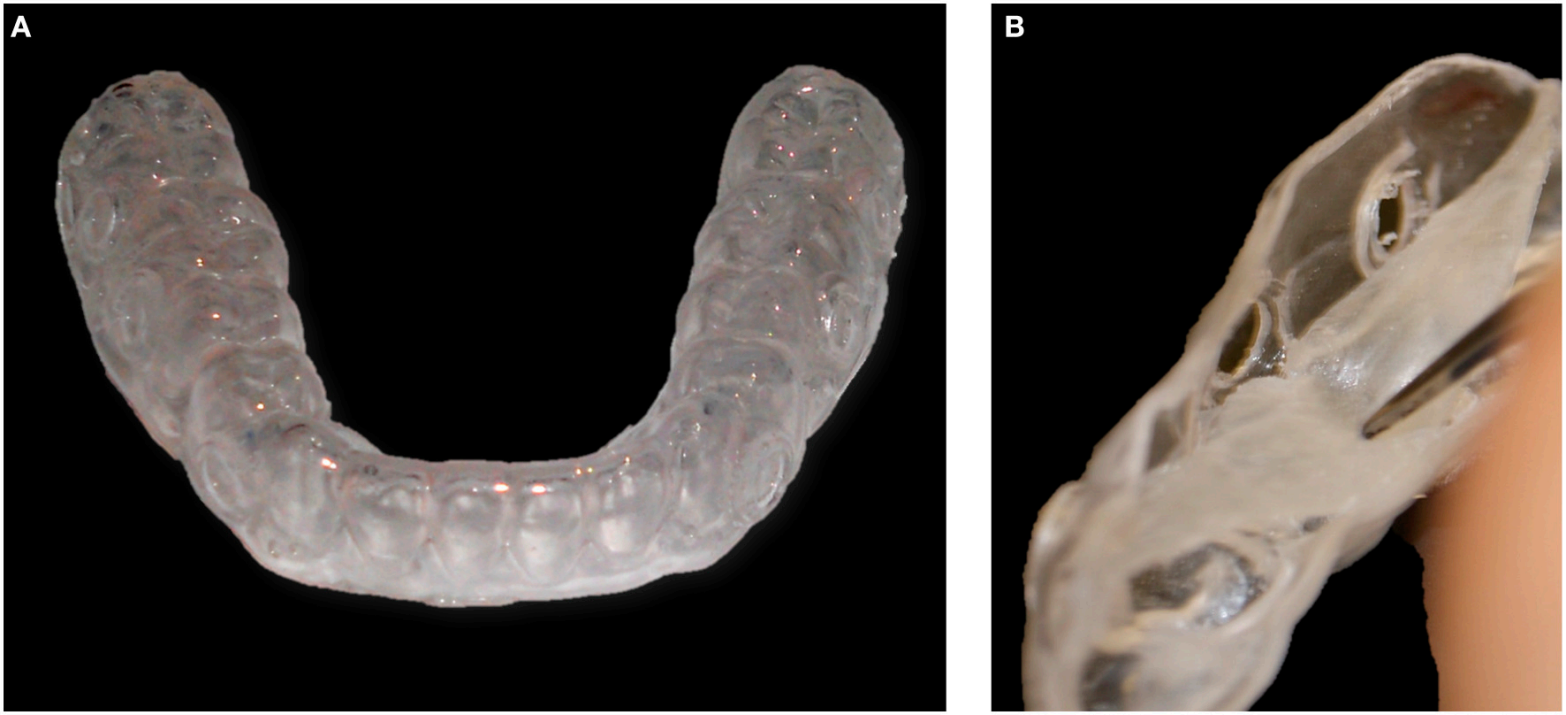
**Intraoral Device of Disk-holding Splints (IDODS). (A)** Frontal view. **(B)** Detail of the pocket where the disks are placed.

On the other hand, buccal devices have been used extensively, allowing the definition of six different groups: AcD (Figures 4A,B) (Nyvad and Fejerskov, 1987a,b, 1989; Nyvad and Kilian, 1987; Hannig, 1997; Netuschil et al., 1998; Hannig, 1999a,b; Fine et al., 2000; Auschill et al., 2001, 2002; Jentsch et al., 2002; Palmer et al., 2003; Scarano et al., 2003; Groessner-Schreiber et al., 2004; Diaz et al., 2006; Chalmers et al., 2007; Hannig et al., 2007b; Scotti et al., 2007; Grossner-Schreiber et al., 2009; Sreenivasan et al., 2009; Azevedo et al., 2012; Rehman et al., 2012; Do Nascimento et al., 2013; Nascimento et al., 2014), LiD (Figures 5A,B; Strassler et al., 1986; Robinson et al., 1997; Wood et al., 1999, 2000, 2002; Arai et al., 2000; Shore et al., 2001; Kato et al., 2004; Robinson et al., 2006; Dong et al., 2010; Kato et al., 2012; He et al., 2013), AcMD (Figures 6A,B; Rimondini et al., 1997; Zucchelli et al., 1998; Giertsen et al., 2000; Zucchelli et al., 2000; Arweiler et al., 2004; Auschill et al., 2004, 2005; Al-Ahmad et al., 2007, 2010a,c, 2013; Dige et al., 2007; Arweiler et al., 2008, 2014; Dige et al., 2009a,b; von Ohle et al., 2010; Bremer et al., 2011), MD (Figure 7; Leonhardt et al., 1995; Simion et al., 1997), TPD (Figure 8; Sennhenn-Kirchner et al., 2007; Al-Ahmad et al., 2009; Sennhenn-Kirchner et al., 2009; Burgers et al., 2010; Gosau et al., 2010; Jung et al., 2010; Claro-Pereira et al., 2011; Brambilla et al., 2012; Gu et al., 2012; Rupf et al., 2012; Hannig et al., 2013b,c; Kensche et al., 2013; Grychtol et al., 2014) and IDODS (Figures 9A,B; García-Caballero et al., 2013; Tomás et al., 2013; Prada-López et al., 2015a,b; Quintas et al., 2015a,b). In the review process, another device was found, a device made completely of silicone used in only one paper (Giordano et al., 2011).

In summary, Tables 2, 3 show all of the papers which studied *in situ* biofilm, classified according to the type of device used. However, this classification was limited by the scant information some authors provided about the devices they used.

**Table 2:** **Main papers which used palatal and lingual devices for the study of ***in situ*** biofilm**.

**Palatal devices**		**Lingual devices**
Benelli et al., 1993	Gameiro et al., 2009	Creanor et al., 1986
Cury et al., 2001	Lima et al., 2009	Macpherson et al., 1990
Hara et al., 2003	Schwarz et al., 2009	Macpherson et al., 1991
Tenuta et al., 2003	Sousa et al., 2009	Jenkins et al., 1988
Auschill et al., 2004^*^	Beyth et al., 2010	Rasperini et al., 1998
Paes Leme et al., 2004	de Mazer Papa et al., 2010	Sreenivasan et al., 2004
Pecharki et al., 2005	Brighenti et al., 2012	Re et al., 2011
Ribeiro et al., 2005	Cochrane et al., 2012	
Korytnicki et al., 2006	Teixeira et al., 2012	
Schwarz et al., 2006	Melo et al., 2013	
Arthur et al., 2007	Pierro et al., 2013	
Schwarz et al., 2007	Bittar et al., 2014	
Scotti et al., 2007^*^	Padovani et al., 2015	
Paes Leme et al., 2008		

* *Palatal and buccal devices were used*.

**Table 3:** **Main papers which used buccal devices for the study of ***in situ*** biofilm, classified by material**.

**Buccal devices**	**Acrylic devices**		**Leeds *in situ* device**	**Acrylic + Metal devices**	**Metal devices**	**Thermoplastic devices**	**IDODS**
Author, year [references]	Nyvad and Fejerskov, 1987a Nyvad and Fejerskov, 1987b Nyvad and Kilian, 1987 Nyvad and Fejerskov, 1989 Hannig, 1997 Netuschil et al., 1998 Hannig, 1999a Hannig, 1999b Fine et al., 2000 Auschill et al., 2001 Auschill et al., 2002 Jentsch et al., 2002 Palmer et al., 2003 Scarano et al., 2003	Groessner-Schreiber et al., 2004 Diaz et al., 2006 Chalmers et al., 2007 Hannig et al., 2007b Scotti et al., 2007^*^ Grossner-Schreiber et al., 2009 Sreenivasan et al., 2009 Azevedo et al., 2012 Rehman et al., 2012 Do Nascimento et al., 2013 Nascimento et al., 2014	Strassler et al., 1986 Robinson et al., 1997 Wood et al., 1999 Arai et al., 2000 Wood et al., 2000 Shore et al., 2001 Wood et al., 2002 Kato et al., 2004 Robinson et al., 2006 Dong et al., 2010 Kato et al., 2012 He et al., 2013	Rimondini et al., 1997 Zucchelli et al., 1998 Giertsen et al., 2000 Zucchelli et al., 2000 Arweiler et al., 2004 Auschill et al., 2004^*^ Auschill et al., 2005 Al-Ahmad et al., 2007 Dige et al., 2007 Arweiler et al., 2008 Dige et al., 2009a Dige et al., 2009b Al-Ahmad et al., 2010a Al-Ahmad et al., 2010c von Ohle et al., 2010 Bremer et al., 2011 Al-Ahmad et al., 2013 Arweiler et al., 2014	Leonhardt et al., 1995 Simion et al., 1997	Sennhenn-Kirchner et al., 2007 Al-Ahmad et al., 2009 Sennhenn-Kirchner et al., 2009 Burgers et al., 2010 Gosau et al., 2010 Jung et al., 2010 Claro-Pereira et al., 2011 Brambilla et al., 2012 Gu et al., 2012 Rupf et al., 2012 Hannig et al., 2013a Hannig et al., 2013b Hannig et al., 2013c Kensche et al., 2013 Grychtol et al., 2014	García-Caballero et al., 2013 Tomás et al., 2013 Prada-López et al., 2015a Prada-López et al., 2015b Quintas et al., 2015a Quintas et al., 2015b

* *Palatal and Buccal devices were used. A Silicone device was used by Giordano et al. (2011)*.

#### Characteristics of buccal devices

The evaluations of each buccal device group according to the ideal characteristics they should meet are presented in Table 4. The AcD and LiD were the buccal devices that showed more limitations than advantages. On the contrary, the TPD and IDODS presented more advantages than limitations.

- Pre-Treatment of Specific Teeth
  The pre-treatment of teeth is only necessary in the LiD group; it needs from etching, bonding and a composite to be glued onto specific teeth (first and second molars).
- Accidental Unsticking
  Accidental loss of samples has been found in the LiD, AcD, AcMD and MD groups. Conversely in TPD and IDODS groups no loss of specimens has been reported.
- Eating and Chewing with the Device
  The LiD allows the user to eat whilst wearing it, without the need for its withdrawal, as happens with AcMD and MD. The AcD, TPD and IDODS do not permit chewing, because they cover the occlusal zone of the molars.
- Withdrawal by the Volunteer
  All of the apparatus, with the exception of the LiD, can easily be withdrawn by the volunteer in order to perform their oral hygiene measures normally (according to the protocol of the study).
- Withdrawal of the Sample by the Investigator
  The withdrawal of the sample from the LiD, AcD and AcMD by the investigator is done with a forceps or a bracket-removing plier. On the other hand, in the TPD, the investigator must take the sample by its backside with tweezers (Burgers et al., 2010; Gosau et al., 2010) or remove the silicone and take the disk by the lateral sides (Jung et al., 2010). In the same way, the IDODS has a specific gap between the two sheets which permits the withdrawal of the disk by the lateral sides.
- Contact with Cheek and Tongue
  The TPD, MD and IDODS have a special framework (with composite in MD) which protects the substrate from making contact with anything other than from the saliva or any other liquids inside the mouth (antiseptic agent). In the AcMD, the disk is located in a sheltered space between the tooth and the device, which protects the biofilm from contact with the cheek and tongue.
- Salivary Flow through the Splint
  Only the IDODS allows salivary flow through it, leaving both sides of the disk exposed.
- Volunteer's Comfort
  AcD are very voluminous, dramatically affecting the phonetics and the esthetics of the volunteers; the same is true for the metal used in the AcMD. Conversely, the LiD (situated in the posterior molar), the TPD and the IDODS (both made of transparent material) are less voluminous. For these reasons, they slightly affect the phonetics and the esthetics.
- Adaptability, Placement and Manufacturing Cost
  Manufacturing processes for these devices are very diverse. All devices except the LiD need a plaster model of the volunteer to be made in order for the device to fit properly. The LiD, being a standard apparatus, does not need any type of individualisation prior to placement. Other devices such as AcD and TPD or the IDODS need a laboratory process, but their fabrication is simple and not too expensive. However, the AcMD and overall the MD (made of Cobalt and Chrome) requires a more complex laboratory process which increases the cost of the device.

In regard to the placement in mouth, there are also differences between the LiD and other devices, due to the fact that it must be placed in the clinic with the proper isolation and specific protocols, similar to those used in bracket bonding. However, all of the other devices are given to volunteers with the instructions for correct use, in the same way as if they were removable orthodontic retainers.

**Table 4:** **Evaluation of the characteristics of buccal devices, as well as the total number of advantages and limitations**.

**Devices**	**Acrylic devices**	**Leeds *in situ* device**	**Acrylic + Metal devices**	**Metal devices**	**Thermo. devices**	**IDODS**	**TOTAL advantages/limitations**
**CHARACTERISTICS**							
Teeth pre-treatment is not necessary	✓	**✗**	✓	✓	✓	✓	5/1
Specific teeth are not necessary	✓	**✗**	✓	✓	✓	✓	5/1
No accidental unsticking	**✗**	**✗**	**✗**	**✗**	✓	✓	2/4
Allows eating	**✗**	✓	✓	✓	**✗**	**✗**	3/3
Easy withdrawal by the volunteer	✓	**✗**	✓	✓	✓	✓	5/1
Easy withdrawal of the sample	**✗**	**✗**	**✗**	**✗**	✓	✓	2/4
No contact with cheek /tongue	**✗**	**✗**	✓	✓	✓	✓	4/2
Allows salivary flow through the splint	**✗**	**✗**	**✗**	**✗**	**✗**	✓	1/5
Allows good oral hygiene	✓	**✗**	✓	✓	✓	✓	5/1
Good esthetic	**✗**	✓	**✗**	**✗**	✓	✓	3/3
Little bulky	**✘**	✓	✓	✓	✓	✓	5/1
Adaptable on the 1st appointment	**✗**	✓	**✗**	**✗**	**✗**	**✗**	1/5
Easy placement at 1st time	✓	**✗**	✓	✓	✓	✓	5/1
Price of the material	+	++	++++	+++++	+++	++++	
**TOTAL advantages/limitations**	5/8	4/9	8/5	8/5	10/3	11/2	

*✓ = The device meets this characteristic*.

***✗** = The device does not meet this characteristic*.

*+ = Relative cost of the manufacturing*.

## Discussion

No previous reference was found to give the authors a base to work with the characteristics of the different devices. Given this circumstance, a list of fourteen important qualities and characteristics was standardized. The fact that the authors have also designed one of the apparatus reviewed could be a potential source of bias. When the list of items was devised the authors tried to abstract from their own design trying to be as “objective” as possible. The items were chosen, based on the authors' experience, for being of capital importance in an “ideal” device. Later, this checklist was modified after completely reading the selected articles in the present review.

The classification of the apparatus was designed prior to the start of the study and subsequently modified in order to clarify its presentation. As the group has previous experience in the field, a first classification based on the material of the device was established. After the data extraction, this classification was extended introducing the position of the substrates.

The review was focused mainly on buccal devices due to fact that both palatal and lingual devices have been less used in the existing literature. In the latter types of devices, the PL-Biofilm was always exposed to contact with the tongue; for this reason, the biofilm would be very disturbed. To prevent this from happening, authors devised protections such as a plastic mesh (in palatal devices) or an artificial gap between the device and the lingual gingiva (in lingual devices). These protections would surely modify the growing environment of the PL-Biofilm, causing possibly, a lack of representativeness of the dental biofilm attached to the enamel surface. On the contrary, these devices permit the development of PL-Biofilm in absence of contact with the oral environment and poor renovation of saliva on its interior, allowing for the growing of biofilm covered with stagnated saliva. This particularity makes them to be useful for the replication of dental caries models. In any case, their use in PL-Biofilm studies is scarce (10 times less than buccal devices). A possible reason for this might be the big influence in phonetics that these devices have.

### Utility of the devices taking into account the microbiological objective of the study

As previously stated, the intraoral devices for the development of PL-Biofilm have been faced to obtain very different results. These range from studying the covering grade of the biofilm, its thickness, bacterial viability or composition before and after applying several antimicrobial agents to the analysis of the effects that the PL-Biofilm itself may have onto a specific substrate. As stated in the material and methods section, the present review was only focused on the use of these devices in order to obtain a PL-Biofilm for a posterior microbiological evaluation. This evaluation has been done by CFUs, electronic microscopes (SEM or TEM), CLSM (after previous staining with dual live/dead fluorochromes or FISH and DAPI for bacterial identification and differentiation). All devices presented good properties in order to use one or another technique. In some cases the necessity of using one or another technique is going to be more related to the type of substrate that it is being used. For CFUs analysis, no troubles were found in any study in terms of collection of the sample, since the sample is harvested normally by vortexing the PL-Biofilm with the substrate (Leonhardt et al., 1995) or by collecting the PL-Biofilm with a cotton pellet directly from the substrate (Korytnicki et al., 2006). For CLSM analysis with previous staining, problems regarding substrates have been referred by some authors (Netuschil et al., 1998; Dige et al., 2007). The use of enamel, and sometimes hydroxyapatite has been related to episodes of autofluorescence of the substrate, increasing the difficulty of differentiation of the background and the sample itself (Netuschil et al., 1998). This can be corrected at the capture time with a specialized software. In any case, substrates that do not produce autofluorescence, such as glass, are preferred when possible, since the less the investigator has to “correct” the image the less biased the technique will be. In the case of using SEM, no troubles were found; with this technique, the sample is prepared for visualization within the substrate. Although no troubles were referred in any study using TEM, some problems may arise when doing the micro-cuts. This may be related to the excess of fragility of some materials. Hydroxyapatite disks due to their conglomerate structure, may shatter when the cut is done. Maybe for this reason, no study using TEM chose this material as a substrate.

### Characteristics of buccal devices

Probably, in an effort to search the most similar substrate to the natural tooth surface, the most commonly used substrate has been the human enamel. Nowadays, other substrates such as titanium (Gosau et al., 2010; Giordano et al., 2011; Do Nascimento et al., 2013) or membranes (Simion et al., 1997; Zucchelli et al., 1998, 2000) are used more frequently in these devices for the study of peri-implantitis or the bacterial colonization in regenerative procedures.

The quantity of the substrates used depends on the design of the apparatus and the specific requirements for the aim of each of the studies. Depending on the latter, some devices are not eligible due to the impossibility of inclusion of enough samples (i.e., a study needing from more than four different samples from the same volunteer cannot be conducted using a LiD).

Scanning and Transmission Electronic Microscopy techniques, traditionally considered as the Gold standard for the visualization of the biofilm (Al-Ahmad et al., 2009), are not the most commonly used techniques in the selected studies. This is probably because of the necessity of altering the biofilm three-dimensional structure and the limitation of the analysis (Hannig et al., 2010). Because of this need of keeping the biofilm unaltered, fluorescence based microscopes, such as the epifluorescence and the CLSM, combined with FISH and DAPI for bacterial identification have been the preferred options. Another technique that has been widely used has been CFU counting, nowadays still considered the Gold standard in bacterial identification (Choudhry, 2016). Despite this, over the last decade, CFU counting has coexisted with other molecular techniques based on the identification of the bacteria by their genome that have questioned the CFUs accuracy (Benítez-Páez et al., 2013), since more than 50% of the bacteria present in the oral cavity are not culturable (Aas et al., 2005).

A wide range of biofilm maturation has been found in this review. Of course, this is a characteristic which is directly related to the aim of the studies. As the devices may be used for long periods of time, the apparatus should be properly designed, not affecting the volunteer's comfort. In the present review, the authors have taken into account that dental biofilm in terms of maturation may be generally considered after 4 h. Before this time, there may possibly exist bacteria adhered to the acquired pellicle. In fact, Hannig et al. (2007a) found bacteria adhered to the acquired pellicle at 3 min; in any case, they were only first colonizers and cannot be considered as a bacterial aggregate which will define a biofilm itself. In the present systematic review, papers analyzing biofilm after 4 h have been considered although in their measures have included any measure before this interval. Nevertheless, we have found authors that analyzed acquired pellicel at 2 h and other authors that analyzed CFUs since the very first 10 min (Leonhardt et al., 1995), or applied FISH tecniques at 30 min (Al-Ahmad et al., 2013).

The pre-treatment of the teeth with etching, bonding/de-bonding procedures and the posterior composite removal could damage enamel. This could result in the production of white-lesions or demineralizations similar to those caused by fixed orthodontia (Artun and Thylstrup, 1986). As the general rule should be to harm the volunteer's integrity as little as possible, devices retained without being adhered to the tooth surface are preferred.

Accidental loss of samples is a problem that has been found in those apparatus (LiD) where disks are attached to the tooth surface. The same situation has been reported in the AcD, AcMD, and MD groups, where specimens are glued or fixed to the device with wax. TPD and IDODS have specific zones where the disks are perfectly retained. Accidentally dropping samples is an important issue, not only for the study, which will lose a specimen, but for medical reasons, due to the potential bronchial aspiration of the disk, which would cause an emergency situation.

A common limitation that applies to most devices is the inability to eat with the apparatus while wearing it. This characteristic would allow the analysis of the biofilm growing in the presence of nutrients coming directly from food. Although, the withdrawal period during meals is brief (15–30 min) and the devices are generally kept in a humid environment, this action implies that the biofilm is not exposed to nutrients or the self-cleansing action of chewing.

The impossibility to remove the LiD by the volunteer hinders the oral hygiene level in the vicinity of the device. Other devices can be removed by the volunteer in order to perform their oral hygiene measures normally (according to the protocol of the study). The ability to brush the teeth makes the use of these devices suitable for volunteers with specific oral diseases, who need to maintain good oral hygiene, such as patients with periodontitis. The study of this biofilm *in situ* could be the ideal method to achieve better knowledge and control of the disease (Marsh, 2005).

The withdrawal of the sample from the LiD by the investigator with a forceps or bracket-removing pliers might disturb the *in situ* biofilm. The same situation is reported when the investigator withdraws the disk from an AcD or AcMD. The design of both TPD and IDODS permit an easy removal of the specimen from the device with tweezers, without disturbing the biofilm.

Another important factor which could disturb the biofilm is contact with the cheeks and tongue during the period when the device is inside the oral cavity. In most apparatus, this issue has been solved with the design of a specific framework that protects the substrate. In the case of the AcMD, the disk is located facing the teeth, avoiding contact with the cheeks. In this specific situation, the growing conditions of the PL-Biofilm are completely different from the real situation given in the buccal teeth surface.

During the oral biofilm formation, the flow of saliva supplies the disk with nutrients (Bowden and Li, 1997). For this reason, if the device encounters a correct salivary flow, the biofilm on the disk will have sufficient nutrients, even though it is contained within the splint. The IDODS was the only device that enabled this salivary flow with a perforation located under every specimen. This salivary flow would not be desirable when studying certain types of biofilms found in places with difficult access, where this flow is limited or even absent (in caries models, for example). To the analysis of that biofilm, it would be necessary to design another type of device which does not permit any salivary flow through the specimen. In the present review, only some palatal and lingual devices (Cury et al., 2001; Tenuta et al., 2003) and one modification of the LiD (He et al., 2013), could be used for this purpose.

Another important criterion that should be taken into account when designing a biofilm device is its effect on the volunteer's comfort. The normal life of subjects is altered by all devices, especially by the bulk of the device and the type of material used, both esthetically and phonetically. If the volunteer's comfort is not affected or is affected as little as possible by the wearing of the device, the volunteer will better fulfill the protocol. If the device is uncomfortable or not esthetic, the volunteers might change their diet or reduce the duration for which the device is placed inside the oral cavity. Every change in their normal life will produce a bias in the growing biofilm. Despite the importance of these parameters, only one study which discussed the volunteer's experience with the device was found after this review (Prada-López et al., 2015b). On the other hand, some papers have registered dropouts by the volunteers during the experiment, because of discomfort (Brambilla et al., 2012) or unclear reasons (Arweiler et al., 2014).

### Device modifications

**Figure 10:**
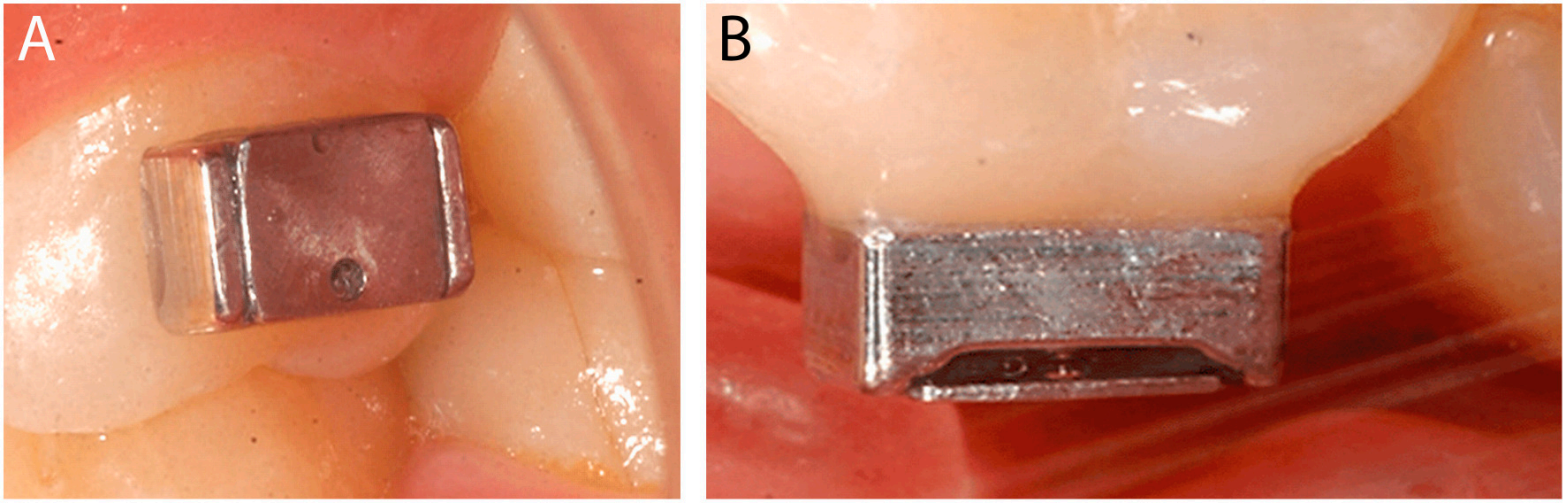
**Modification of the Leeds ***in situ*** device (by He et al., 2013 in Plos One). (A)** Intraoral buccal view. **(B)** Detail of the gap between the two metal slides where the biofilm grows.

Finally, it is important to highlight that some authors made small variations from the prototype. For instance, in 2012, Gu et al. (2012) added an orthodontic wire to the TPD with the aim of making it more resistant. In the same way, and in order to improve the LiD design, He et al. (2013) used an additional metal sheet to protect the biofilm from contact with the cheek (Figures 10A,B); eventually, this modification meant that the biofilm grew between two metals, without taking into account the electric covalent flows produced between two face-to-face metal sheets, which would undoubtedly affect the development of the biofilm.

### Ideal model for PL-biofilm development

After this deep review of the existent literature, the authors would like to show their own perspective of the “ideal” *in situ* model for PL-Biofilm development. In the authors' opinion, the ideal device should meet at least all the previously stated ideal characteristics (Table 1). After their analysis, it seems clear that it should be made of a transparent material (polyethylene, silicone…) for esthetic reasons. It should not be glued on the surface of the teeth in order not to damage them in any extent. In addition, the device should be designed for disk accommodation without gluing; this will avoid accidental loss of specimens. Moreover, the device should permit removal from the oral cavity to allow the volunteer to perform their daily oral hygiene measures. Furthermore, it would be desirable that the apparatus was designed to facilitate the salivary flow through it, simulating the interproximal spaces. All these, and other characteristics, are only fulfilled by the IDODS, being the one that is closest to the “ideal” model. On the other hand, there are other aspects that should be improved in its design: the impossibility to eat with it and the need of more than one appointment for its adaptation. Its design should evolve into a model which does not cover the occlusal faces of the teeth (permitting eating while wearing it). The vestibular band of the IDODS could be used instead of all apparatus. This would allow that the occlusal surfaces of the teeth would not be covered anymore. Other ways of retention, apart from self-retention of the teeth or gluing would be possible. Using orthodontic wires or rubbers may help with this. At the same time, this would permit device removal from the mouth by the volunteer for oral hygiene measures, but also the possibility to eat with them to get closer to the clinical reality of the tooth surfaces. This modification could also help in their adaptability due to the fact that only the vestibular part of the hemi-arch would be used. Consequently, it might allow the design of a standard vestibular part of the apparatus (maybe three different sizes) avoiding the need of more than one appointment for its adaptation.

### Future investigations

The majority of the published papers have not described the device or the manufacturing methodology properly. In these cases, reproduction of any device would be more difficult, and standardization would be impossible. Consequently, the potential to compare results between studies or to apply the same methodology would be a utopian situation. For this reason, a specific description would be very useful when any groups subsequently design new devices, as previously included by some authors (Robinson et al., 1997; Sreenivasan et al., 2009; Tomás et al., 2013; Prada-López et al., 2015b; Quintas et al., 2015b).

In addition, no papers could be identified which have compared the characteristics of the device-formed-biofilm positioned at buccal (PL-Biofilm) with the tooth-formed-biofilm (dental plaque). From the authors' point of view, the quality or the relevance of the PL-Biofilm should be the primary issue of every study on oral biofilm. When using a device in order to assess *ex vivo* the biofilm formed *in situ*, the first question that a researcher should pose would be if the biofilm formed in the artificial substrate is representative of that characteristic to be studied (biofilm in a caries lesion, biofilm in the interproximal area, biofilm in the vestibular area…). Unfortunately, not a single study has proved this until the moment. To overcome this limitation, our group has designed a study for testing the bacterial viability and composition of the PL-Biofilm, comparing at the same time with the contralateral teeth in a split-mouth design. This will permit to evaluate and validate the biofilm formed on the substrates carried in the IDODS compared to that naturally formed in the surface of the teeth (unpublished data). This comparison would confirm the correct validation of the device and provide more evidence of its applicability to the study of the oral biofilm *in situ*. Previously, following this idea, Creanor et al. (1986) analyzed the CFUs on a lingual device and compared them to those found in natural dental surface, but only in one volunteer. They found that the microbial composition of their PL-Biofilm was relatively consistent compared the natural plaque, although the latter showed more variation.

In the present review, only one study which used self-perception questionnaires was identified. Prada-López et al. (2015b) used a Likert-type questionnaire to evaluate the influence of the device in the esthetics, hygiene, comfort and complication of withdrawal. The questionnaire showed really acceptable results of the influence of the IDODS on the volunteer's life. For further investigations, a questionnaire which measures the influence of the device on the normal daily life of the volunteer would be useful, as it would provide a more accurate vision of the volunteer's comfort whilst wearing these devices.

## Conclusion

Buccal devices were the most commonly used for the study of *in situ* biofilm. The majority of buccal devices seemed to slightly affect the volunteer's comfort, the IDODS being the closest to the “ideal” model. However, there are other aspects that should be improved in its design: the impossibility to eat with it and the need of more than one appointment for its adaptation.

Papers should include more information about manufacturing their devices. Therefore, any new device must consider the limitations of the previous ones, paying particular attention to the needs of the volunteer and the biofilm formation. In addition, all of the devices must test the volunteer's experience and the microbiological differences between device-formed biofilm and tooth-formed biofilm and studies should include a feedback exercise.

## Author contributions

IP, VQ Conception and design of the review, revising of the literature, acquisition, and analysis of data and drafting the article. CV Making different devices for obtaining figures and revising of the literature. DS, IT Conception and design of the review, revising of the literature and manuscript and final approval.

## Funding

This work was supported by project EM2014/025 from Regional Ministry of Culture, Education and University (regional government of Galicia, Spain), which is integrated in Regional Plan of Research, Innovation and Development 2011–2015. The funders had no role in study design, data collection and analysis, decision to publish, or preparation of the manuscript.

### Conflict of interest statement

The authors declare that the research was conducted in the absence of any commercial or financial relationships that could be construed as a potential conflict of interest.

## Abbreviations

AcD: Acrylic Device
AcMD: Acrylic and Metal Device
CFU: Colony Forming Units
CLSM: Confocal Laser Scanning Microscope
DAPI: Diamidino-2-Phenylindole
FISH: Fluorescence *in situ* Hybridisation
IDODS: Intraoral Device of Overlaid Disk-holding Splints
LiD: Leeds *in situ* Device
MD: Metal Device
PICO: Patient/Population Intervention Comparsion Outcome
PL-Biofilm: Plaque-Like Biofilm
TPD: Thermoplastic Device.
